# Optimization of gene editing in cowpea through protoplast transformation and agroinfiltration by targeting the phytoene desaturase gene

**DOI:** 10.1371/journal.pone.0283837

**Published:** 2023-04-05

**Authors:** Aya Bridgeland, Sudip Biswas, Nikolaos Tsakirpaloglou, Michael J. Thomson, Endang M. Septiningsih

**Affiliations:** Department of Soil and Crop Sciences, Texas A&M University, College Station, TX, United States of America; International Institute of Tropical Agriculture, KENYA

## Abstract

Cowpea (*Vigna unguiculata*) is a legume staple widely grown across Sub-Saharan Africa and other tropical and sub-tropical regions. Considering projected climate change and global population increases, cowpea’s adaptation to hot climates, resistance to drought, and nitrogen-fixing capabilities make it an especially attractive crop for facing future challenges. Despite these beneficial traits, efficient varietal improvement is challenging in cowpea due to its recalcitrance to transformation and long regeneration times. Transient gene expression assays can provide solutions to alleviate these issues as they allow researchers to test gene editing constructs before investing in the time and resource- intensive process of transformation. In this study, we developed an improved cowpea protoplast isolation protocol, a transient protoplast assay, and an agroinfiltration assay to be used for initial testing and validation of gene editing constructs and for gene expression studies. To test these protocols, we assessed the efficacy of a CRISPR-Cas9 construct containing four multiplexed single-guide RNA (sgRNA) sequences using polyethylene glycol (PEG)-mediated transformation and agroinfiltration with *phytoene desaturase* (*PDS*) as the target gene. Sanger sequencing of DNA from transformed protoplasts and agroinfiltrated cowpea leaves revealed several large deletions in the target sequences. The protoplast system and agroinfiltration protocol developed in this study provide versatile tools to test gene editing components before initiating plant transformation, thus improving the chance of using active sgRNAs and attaining the desired edits and target phenotype.

## Introduction

Since the advent of CRISPR-Cas9 technology, genome editing techniques and protocols have become largely optimized for major crops such as rice, wheat, and maize [1–4]. However, there remains a gap in optimization for most other crop species, including cowpea. Cowpea (*Vigna unguiculata*), is a high-protein, nutritionally dense annual legume species that is grown as a staple crop across semi-arid regions of Africa, Asia, and South America [5–7]. Although cowpea has previously been considered an orphan crop due to a lack of scientific attention, its superior traits, such as drought tolerance, nitrogen-fixing capabilities, and ability to grow in marginal soils, have brought it newfound consideration as an archetype of climate-resilience and sustainability [5, 7]. Despite its many advantages as a crop against abiotic stresses, cowpea still suffers from low productivity and large yield gaps due to biotic susceptibility to pests, especially insects [8–10]. Historically, cowpea varietal improvement has been challenging due to a low-diversity breeding population and sexual incompatibility with wild relatives [8, 11–13]. The recent release of a Maruca pod borer-resistant variety, developed via the transgenesis of a *Bacillus thuringiensis* gene with an *Agrobacterium* vector, shows that a biotech-based approach can successfully drive crop improvement in ways that were not possible using conventional means [14]. Nonetheless, only limited CRISPR-Cas9 genome editing applications have been established in cowpea thus far, based on hairy roots and embryonic axis explant transformation [15–17].

Like other legumes, cowpea is notoriously recalcitrant to transformation, which proves to be a major bottleneck in crop improvement. Even in successful instances, transformation frequency is low, and the long regeneration times make attempting a transformation experiment a high-investment activity [18, 19]. For these reasons, a major priority of cowpea research is to improve these bottleneck areas, and one of the examples is by developing transient gene expression assays for the rapid testing of gene editing constructs. In this regard, protoplast transformation and leaf infiltration were used as transient assay platforms in this study. Protoplasts are often used as a single-guide RNA (sgRNA) activity validation step, as it allows the transformation and sequencing of live cells with a quick turnaround time. Using protoplasts, any number of factors, including promoter type, plasmid size, and sgRNA activity, can be tested, increasing the odds of success in actual transformation events [20, 21]. Protoplasts have been used for the testing of CRISPR/Cas9 constructs and sgRNA activity in a diverse range of species, including legumes such as peanut and chickpea, but a protocol has not yet been developed for cowpea [22–24]. Likewise, Agroinfiltration of leaves has also been used with the CRISPR-Cas9 system for sgRNA validation and gene expression assays [25–27]. The current study aims to knock out the cowpea *phytoene desaturase* (*PDS*) gene using a CRISPR-Cas9 construct designed with four multiplexed sgRNAs through PEG transformation in cowpea protoplasts and agroinfiltration in cowpea leaves.

## Materials and methods

### Plant material

Cowpea seeds of variety IT97K-499-35 were used in this study [8]. Seeds were multiplied in the greehouse facility at Texas A&M University (College Station, TX). Plants were grown in pots containing soils with day/night temperatures of 32/26°C and a 16/8 h light/dark cycle.

### Protoplast isolation by the leaf-cutting method and tape sandwich method

The leaf-cutting method was tested following the protocol by Wu and Hanzawa et al. 2018 [28] with the following modifications (S1 Fig, upper panel). Primary leaves from 6-day-old cowpea plants were cut from seedlings, sliced into ~0.5 mm strips after removing the midrib, and then placed in the enzyme solution (S1 Table). Two leaves were used per 10 ml of enzyme solution. The strips were vacuum infiltrated for 15 min and incubated under low-light conditions for 5.5 h with gentle shaking (45 *rpm*).

The tape sandwich method was tested by following the Wu et al. 2009 protocol [29] with the following modifications (S1 Fig, lower panel). The upper epidermal surface of a 7-day/12-day-old cowpea primary leaf was fixed to a piece of Fisherbrand Colored Labeling Tape. A piece of 3 M Magic Tape (standard clear office tape) was gently pressed to the lower epidermal surface, then peeled off to remove the lower epidermal surface layer. The leaves (with the Fisherbrand tape still attached) were transferred to a beaker with enzyme solution (S1 Table) to a ratio of 10 ml solution to 1 g leaf tissue. Leaves were vacuum infiltrated for 10 min and then shaken at 45 *rpm* for 1.5 h. After this initial incubation, the enzyme solution was poured off, and fresh enzyme solution was added to the leaves. The incubation was then continued for an additional 3 h at 45 *rpm*.

After the incubation for both methods, the enzyme solution was poured into a 50 ml Falcon tube, and an equivalent amount of W5 solution (S1 Table) was added to stop the digestion. The solution was poured through a 40 μM nylon mesh to remove large pieces of debris, then centrifuged at 100 x *g* for 2 min at room temperature. The supernatant was removed, and each tube was resuspended with an equivalent amount of W5 solution.

Protoplasts were counted on a hemocytometer. The number of protoplasts in 4 squares of the hemocytometer grid were counted to calculate protoplast concentration. The protoplast density was then calculated as follows: protoplast number (per gram of tissue) = the average count of protoplast per square × 10^4^.

### PEG-mediated protoplast transfection

Following the successful isolation of protoplasts, the scaled-up protoplast transfection method was performed as adapted from the protocols of Wu and Hanzawa et al. 2018 and Li et al. 2011 [28, 30]. The 15 ml Falcon tubes were coated with 5% (w/v) fetal bovine serum, then spun down at maximum speed for 1 min, and the excess serum was removed. Next, 60 μg of plasmid DNA (pDNA) was added to each tube while keeping one tube as a negative control with no pDNA added. A total of 400 μl of protoplasts with a concentration of 2.5 x 10^5^ protoplasts/ml were added to the tube and pipetted gently to mix with the pDNA. Subsequently, 460 μl of freshly prepared 40% PEG-4000 solution (S1 Table) was added to each tube and pipetted gently to mix. The tube was incubated at room temperature for 30 min. Finally, 3 ml of W5 solution was added to stop the transformation, and the tubes were centrifuged at 250 x *g* for 1 min at room temperature. The supernatant was removed without disturbing the protoplast pellet. Protoplasts were then resuspended with 200 μl of W1 solution (S1 Table). Tubes were covered in aluminum foil and kept at 4°C for 72 h until DNA was extracted.

### sgRNA design for *VgPDS* and *in vitro* ribonucleoprotein assay

Single-guide RNAs (sgRNAs) from the outputs of both CRISPR-P 2.0 and CRISPRdirect sgRNA design tools [31, 32] were selected to target the *VgPDS* gene. The DNA sequence of the cowpea *PDS* gene was obtained from the Legume Information System (LIS) database (www.legumeinfo.org) and was used as the input in the programs for the sgRNA design. Four sgRNAs (sgRNA1: CCGGCAATAACGACCTTCAA**CGG**, sgRNA 2: CTTCAGTTCGTGCTTCTAAG**AGG**, sgRNA 3: GAAGCTAGAGACGTTCTAGG**TGG**, and sgRNA 4: ATATGTGTCTGGCGCCAAGC**TGG**) were designed from the outputs of the two programs and synthesized at Synthego, Inc.

Genomic DNA (gDNA) was extracted from young cowpea using the SDS method, following a Cetyl Trimethyl Ammonium Bromide (CTAB) protocol based on the publications by Doyle and Doyle 1987 [33]. The region of the *PDS* gene containing the sgRNA sequences was genotyped (primers used listed in S2 Table) and amplicons were purified using the Qiagen QIAquick Gel Extraction Kit (Hilden, Germany). TOPO-cloning followed by Sanger sequencing (Eurofins Genomics LLC, Louisville, KY) confirmed that the sgRNA target sequence in our cultivar was identical to the one obtained from the LIS database. Additionally, purified PCR fragments were used for an *in vitro* ribonucleoprotein assay to validate the designed sgRNAs following the protocol “*In vitro* digestion of DNA with Cas9 Nuclease, *S*. *pyogenes* (M0386)” from New England Biolabs (Ipswich, MA, USA) with a few modifications. In this case, a 27 μL reaction mixture containing 30 nM of synthesized sgRNA, 30 nM of Cas9 nuclease, and 3 μL of 10× NEB buffer 3.1 were pre-incubated for 10 min at 25°C. Afterward, 100 ng substrate purified PCR product was added to make a total reaction volume of 30 μl and incubated at 37°C for 1 h. After adding 1 μl of proteinase K, the reaction mixture was kept for 10 min at 56°C, and fragment analysis was then performed using a 2% (g/v) gel electrophoresis.

### Plasmid preparation and constructs

Golden Gate Cloning was used to develop the *VgPDS*-targeting plasmid encoding the Cas9 enzyme and sgRNA cassette following the publication by Čermák, et al. 2017 [34]. For these, three intermediate module plasmids A, B, and C were prepared for the construction of the CRISPR-Cas9 vector. For module A, pMOD_A0101 (Addgene plasmid #90998) was used where AtCas9 was expressed (*Arabidopsis*-codon optimized SpCas9) by a Cauliflower Mosaic Virus 35S (CaMV 35S) promoter. The pMOD_B2303 vector was used for module B. The polycistronic csy4 system [35] was used for the expression of multiple sgRNAs and the cassette containing the four sgRNA sequences for *VgPDS* was synthesized (Macrogen) and placed in a pUC57 intermediate vector (Genscript Biotech Ltd., Piscataway, NJ, USA), then was digested in-house using restriction enzymes (*Pst*I and *Xho*I) and placed in the pMOD_B2303 vector using T4 Ligase (NEB) following the manufacturer’s recommendations. Expression of the sgRNA cassette was driven by the CmYLCV (Cestrum Yellow Leaf Curling Virus) promoter. The pMOD_A0101, modified pMOD_B2303, and empty vector pMOD_C0000 (Addgene #91081) were assembled into a non-binary vector, pTRANS_100 (Addgene #91198) and binary vector pTRANS_210 (Addgene plasmid # 91108) by Golden Gate protocol using the *Aar*I enzyme. Two vectors were developed: a binary vector (pTRANS_210-*VgPDS*) to be used for *Agrobacterium* transformations and a non-binary vector (pTRANS_100-*VgPDS*) to be used for protoplast transformations.

### Mutation analysis for the protoplast assay

At four days post-transfection in dark conditions, the cowpea protoplasts were collected by centrifugation at 13,000 *rpm*, and genomic DNA was then extracted with the CTAB protocol [33]. The Cas9–sgRNAs target sites of DNA segments were amplified with Phusion polymerase using pairs of allele-specific primers (S2 Table). Gradient PCR was performed with an initial denaturation step of 98°C for 30 s, followed by 32 cycles of 98°C for 30 s, 55°C– 58°C for 30 s, and 72°C for 30 s, and a final extension of 72°C for 7 min. The PCR product was then purified by gel extraction and cloned into Topo vector (Thermofiser Scientific, Waltham, MA). Any mutations in the *VgPDS* target site were characterized by sanger sequencing.

### Agroinfiltration assay

Binary vectors (CmYLCV_promoter_:GUS, 35s_promoter_:GUS vector and pTRANS_210-*VgPDS*) were transferred into *A*. *tumefaciens* strains EHA105 and AGL-1 via the freeze-thaw transformation method [36] A single colony of *Agrobacterium* strains containing the desired plasmid was inoculated into liquid LB media with specific antibiotics (Rifampicin was used for EHA105, rifampicin and carbenicillin for AGL-1 agrobacterium strain selection, and kanamycin was used for all vector selection). Cultures were put in a shaker incubator overnight at 28°C at 250 *rpm*. Bacteria were pelleted by centrifugation (4,000 *g* for 30 min) and resuspended to an OD_600_  =  1.0 in bacterial resuspension medium (1/2 MS, 3% sucrose, 10 mM MgCl_2_, 200 μM acetosyringone, pH 5.8). Bacterial suspensions were delivered into the underside of leaves of 7- day-old plantlets using a blunt-tipped 1 ml plastic syringe by applying gentle pressure. The infiltrated leaves were harvested three days later. GUS assay was performed in cowpea leaves that were infiltrated with *A*. *tumefaciens* strains EHA105 and AGL-1 containing CmYLCV_promoter_:GUS and 35s_promoter_:GUS vector [37]. Genomic DNA was isolated from the leaves infiltrated with *A*. *tumefaciens* strain EHA105 containing binary vector (pTRANS_210-*VgPDS*), and any mutations in the *VgPDS* target site were characterized by PCR and sanger sequencing.

## Results

### High-efficiency protoplast isolation by the tape sandwich method

**Fig 1:**
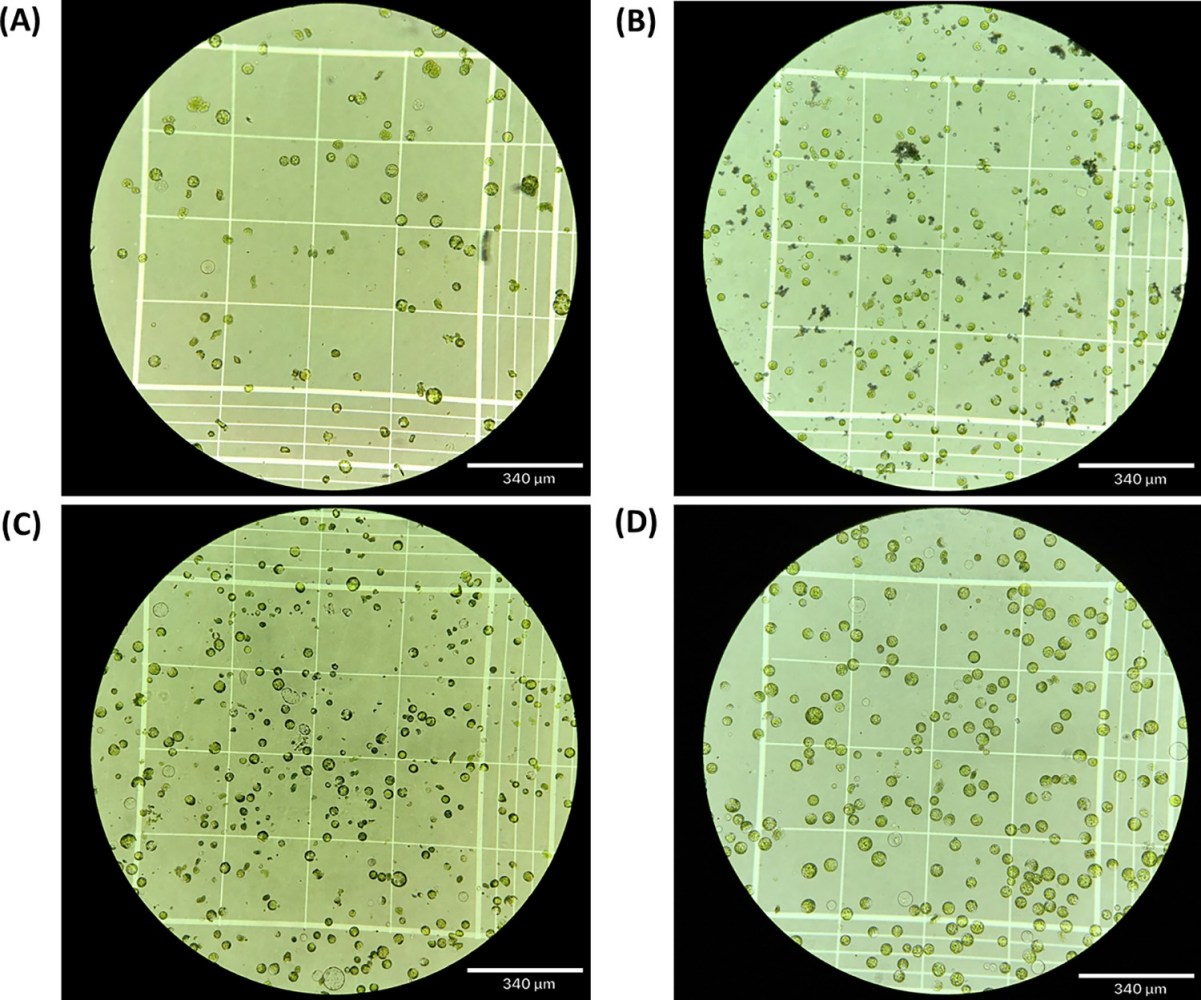
Comparison of protoplast isolation from leaf cutting method versus tape sandwich methods. (A) Protoplasts isolated via the original leaf-cutting method. (B) Protoplasts isolated via the tape sandwich method from 7-day-old plants without optimization. (C) Protoplasts isolated via the tape-sandwich method from 7-day-old plants with optimization. (D) Protoplasts isolated via the tape-sandwich method from 12-day-old plants with optimization.

Optimizing the exposure of mesophyll surface area is critical to obtain high numbers of protoplasts from young leaves. Our results showed that the tape method was more efficient than the leaf-cutting method for preparing leaves for the enzyme solution (Table 1, Fig 1). The leaf-cutting method isolated approximately 175,000 protoplasts per gram of starting tissue (protoplasts/g), with the total time to isolation taking approximately 7 h, while the tape sandwich method isolated 200,000 protoplasts/g in 3 h (Table 1 and Fig 1). The time to isolation also included the front-end work, such as making enzyme solution and preparing the leaves, as well as post-enzyme incubation steps, such as counting protoplasts on the hemocytometer and the 30 min incubation on ice.

**Table 1:** Comparison of protoplast isolation methods.

Method Description	Tissue (days)	Time (h)	Average Yield (protoplasts/g)	Debris
**Leaf cutting**	6–7	7	~175,000	Medium
**Tape sandwich**	6–7	3	~200,000	Low
**Optimized tape sandwich**	6–7	5	~1,675,000	Low
**Optimized tape sandwich**	10–12	5	~7,550,000	Very Low

Different incubation conditions and different ages of plants were tested to optimize the tape sandwich method. The incubation conditions of the Hibi et al. 1975 method [38] added an extra 30 min preliminary incubation step and two additional hours relative to the unmodified tape sandwich method (Table 1). Protoplast yield was significantly higher when using the longer, two-stage incubation, yielding approximately 1,675,000 protoplasts/g compared to 200,000 protoplasts/g from the shorter incubation. On the other hand, protoplasts isolated from 12-day-old plants yielded 7,550,000 protoplasts/g, significantly higher than 6-7-day-old plants. In addition, it was observed that the enzyme/protoplast solution from 12-day-old plants had much less debris than that from 6-7-day-old plants (Table 1). Hence, the optimized tape sandwich method was highly consistent, and each application of this methodology in subsequent trials produced large numbers of protoplasts, sufficient for dozens of transformations.

**Fig 2:**
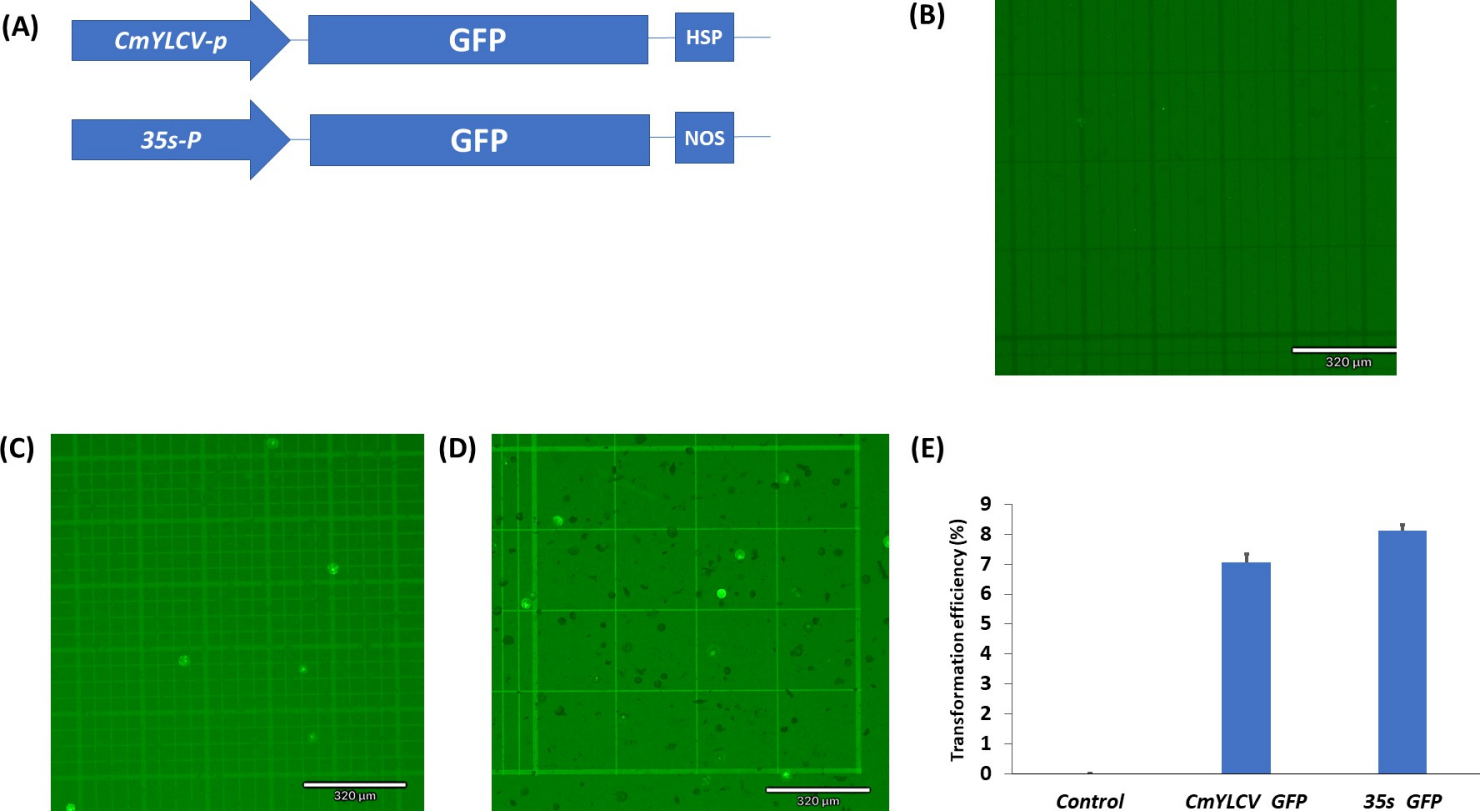
GFP expression under CmYLCV and 35S promoter in cowpea protoplasts. (A) CmYLCV_promoter_:GFP and 35S_promoter_:GFP vectors used in this study. (B) Control protoplasts (no GFP plasmid). (C) Protoplasts with GFP expression under CmYLCV promoter. (D) protoplast with GFP expression under 35S promoter. (E) The transformation efficiency (TE) of protoplasts transformed with CmYLCV:GFP and 35S:GFP plasmid. The protoplasts TE was evaluated after incubation in 40% PEG solution. Values represent means ± SE (n = 7).

We also tested the activity of the two constitutive promoters, 35S and CmYLCV, in cowpea protoplasts. Transformation efficiency was calculated as follows: (the number of protoplasts expressing GFP/total number of protoplasts) × 100. We found that protoplasts transformed with 35S:GFP gave slightly higher transformation efficiency than CmYLCV:GFP based on the number of GFP expressed protoplasts (Fig 2). Temperature, PEG and plasmid concentration play a crucial role in protoplast transformation. In this study, we found the highest transformation efficiency at 40% PEG, 60 μg plasmid concentration and 13°C for 24 h.

### *In Vitro* digestion of *VgPDS* targets with sgRNA and Cas9

**Fig 3:**
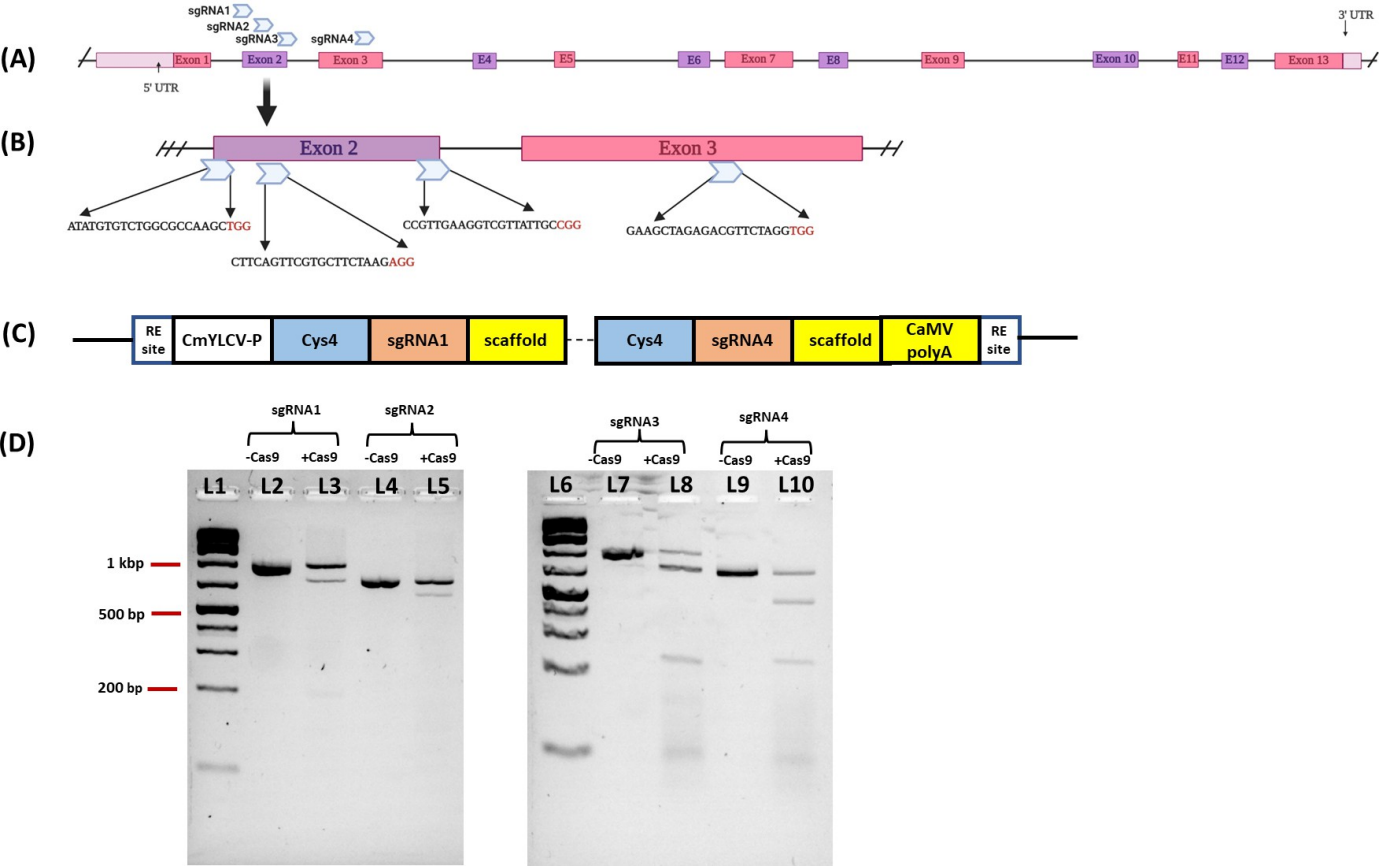
Details of the cowpea *phytoene desaturase* (*PDS*) gene. (A) Schematic representation of cowpea *VgPDS* gene. (B) Target region of *PDS* gene where four sgRNAs were designed. (C) Schematic diagram representation of cys4–sgRNAs cassette targeting the *VgPDS* gene. (D) *In vitro* digestion of *VgPDS*. Lanes L1 and L6: 1 kb Plus ladder, L2: uncut *VgPDS* target region, L3: *VgPDS* target region digested with Cas9 and sgRNA1 (expected bands of 753 bp and 218 bp), L4: uncut *VgPDS* target region, L5: *VgPDS* target region digested with Cas9 and sgRNA2 (expected bands of 648 bp and 130 bp), L7: uncut *VgPDS* target region, L8: *VgPDS* target region digested with Cas9 and sgRNA3 (expected bands of 741 bp and 230 bp), L9: uncut *VgPDS* target region, L10: *VgPDS* target region digested with Cas9 and sgRNA4 (expected bands of 524 bp and 254 bp) (partially designed with BioRender.com, accessed on 10 December 2022). Original gel images are provided as S1 Raw images.

*In vitro* ribonucleoprotein (RNP) assay for the four sgRNAs targeting a PCR amplicon flanking the target site of the cowpea *PDS* gene was performed using the RNP complexes with purified Cas9 (Invitrogen, Waltham, MA, USA) and synthetic gRNAs (Synthego, Redwood City, CA, USA). The negative controls had uncut PCR products, while three bands were seen for the cut amplicon with sgRNA1, sgRNA2, sgRNA3 and sgRNA4, indicating that all sgRNAs efficiently cut their target nucleotide sequences in the *VgPDS* gene targets (Fig 3).

**Fig 4:**
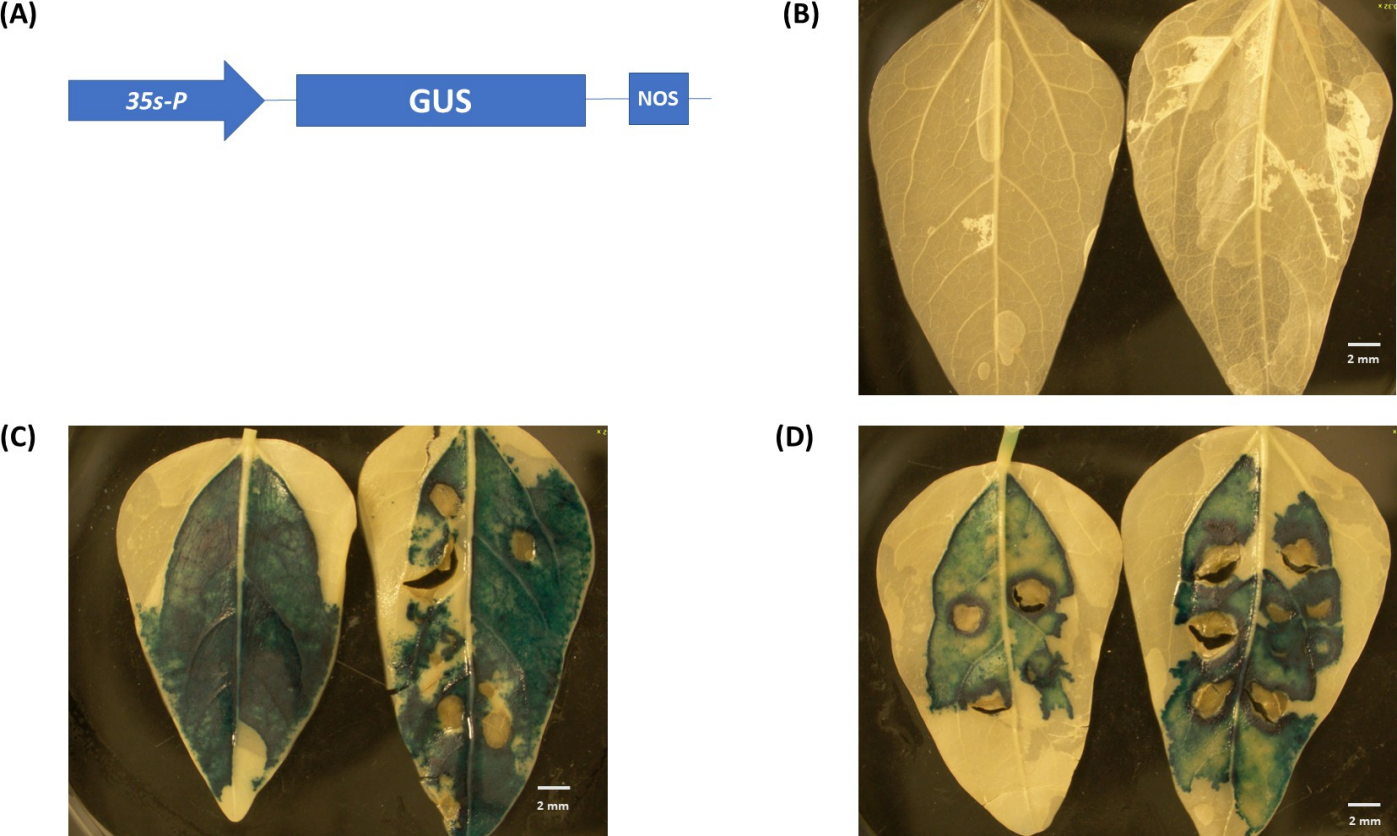
Transient GUS expression *via* agroinfiltration with 35S_promoter_:GUS construct in cowpea leaves. **(**A) 35S_promoter_:GUS construct used in this study. (B) Control leaves (infiltration with *Agrobacterium* strain EHA105 without vector). (C) Cowpea leaves infiltration with *Agrobacterium* strain EHA105 containing 35S_promoter_:GUS vector. (D) Cowpea leaves infiltration with *Agrobacterium* strain AGL-1 containing 35S_promoter_:GUS vector.

**Fig 5:**
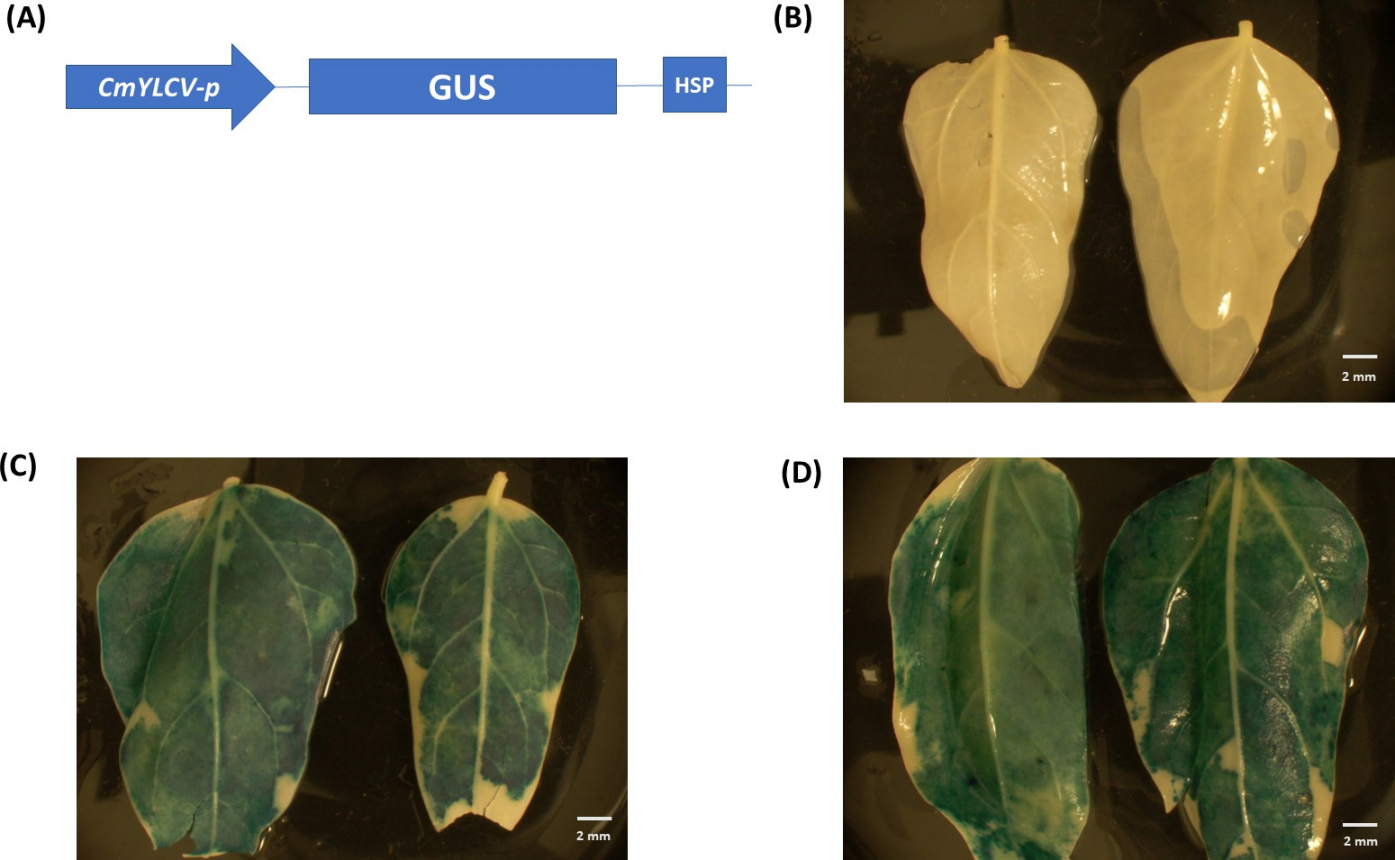
Transient GUS expression *via* agroinfiltration with CmYLCV_promoter_:GUS vector in cowpea leaves. (A) CmYLCV_promoter_:GUS construct used in this study. (B) Control leaves (infiltration with *Agrobacterium* strain AGL-1 without vector). (C) Cowpea leaves infiltration with *Agrobacterium* strain EHA105 containing CmYCLV_promoter_:GUS vector. (D) Cowpea leaves infiltration with *Agrobacterium* strain AGL-1 containing CmYLCV_promoter_:GUS vector.

We conducted agroinfiltration experiments using unifoliate leaves of greenhouse grown 7-10-day-old cowpea plants under laboratory conditions. Our results showed that the transient expression of the GUS reporter worked well under these conditions (Figs 4 and 5). We tested the expression of a GUS reporter under the 35S and CmYLCV promoter separately. Two different Agrobacterium strains (EHA105 and AGL-1) were used in this study. Our results showed that when using the 35S promoter, the EHA105 strain produced higher GUS expression than the AGL-1 strain (Fig 4). However, the CmYCLV promoter overall gave higher GUS expression than the 35S promoter for both Agrobacterium strains (Fig 5).

### Editing of the *VgPDS* gene in cowpea protoplasts

**Fig 6:**
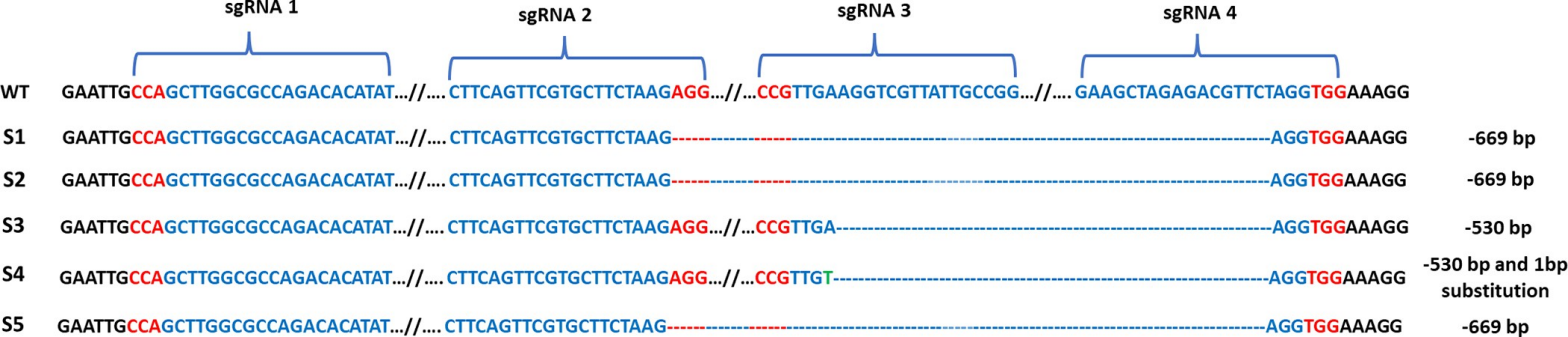
Sanger sequencing results of the *VgPDS* gene derived from cowpea protoplast after PEG mediated transformation. sgRNA 1, sgRNA 2, sgRNA 3 and sgRNA 4 positions are indicated. PAM sequences are highlighted in red letter. WT indicates wild type that was not edited. Dash lines (**—**) are the deleted nucleotides.

To test the gene-editing efficacy of the CRISPR-Cas9 vector for cowpea *VgPDS*, Cowpea protoplasts were transformed with CRISPR-Cas9_*VgPDS*_sgRNAs vector by our optimized PEG mediated transformation protocol. Genomic DNA was extracted to amplify the DNA fragment containing the target site. Sanger sequencing of targeted PCR products obtained from the isolated genomic DNA of each protoplast pool was used to detect the editing efficiency and patterns. The sequencing results revealed various large deletion mutations which covered sgRNA2, sgRNA3 and sgRNA4. We identified 672 bp deletions in three samples (P1, P3 and P4) and 681 bp deletions in two samples (P2 and P5) (Fig 6). However, we did not find any mutation in the sgRNA1 region.

### Editing of the *VgPDS* Gene in cowpea leaves by agroinfiltration

**Fig 7:**
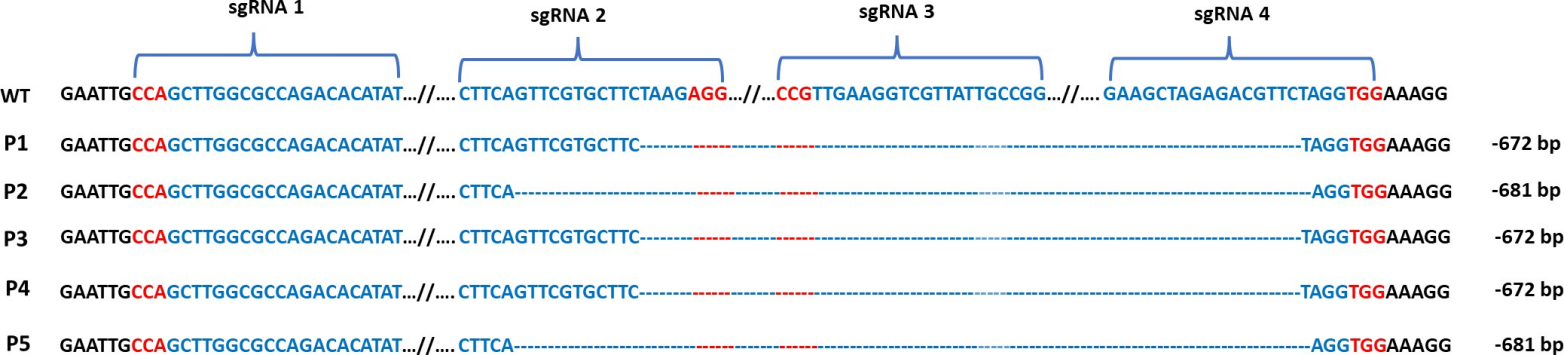
Sanger sequencing results of *PDS* gene derived from agroinfiltrated cowpea leaves. The sgRNA1, sgRNA2, sgRNA3 and sgRNA4 positions are indicated. PAM sequences are highlighted in red letter. WT indicates wild type that was not edited. Dash lines (**—**) are the deleted nucleotides and one substitution bp shows as a green letter in S4.

We also tested the editing efficiency of CRISPR-Cas9 vector for cowpea *VgPDS* by agroinfiltration. After seven days of agroinfiltration with Agrobacterium strain EHA105 containing CRISPR-Cas9_*VgPDS*sgRNA, we isolated the DNA from the infiltrated cowpea leaves. Any mutations of the target *VgPDS* sequencing were detected by sequencing. Similar to the protoplast editing results, we also identified large deletions in the *VgPDS* sgRNA regions. We found 669 bp deletions in three samples (S1, S2 and S5), 530 bp deletion in one sample (S3), and 530 bp deletion and 1 bp substitutions in one sample (S4) (Fig 7).

## Discussion

The protoplast isolation protocol developed through this study combines simple procedures, high yield, reproducible results, and a moderate experimental run time. Through this study, we have demonstrated that the success of cowpea protoplast isolation mainly depends on the method to expose the mesophyll area, the age of plant tissue, and the incubation conditions. Specifically, results indicate that the combination of complete removal of the lower epidermis,10-12-day-old plants and specific incubation times are crucial factors in establishing a method that is both high-yielding and performs consistently well. Likely the most crucial parameter in the optimization process was testing how to remove the lower epidermis of the primary leaves without causing damage. Maximizing the mesophyll surface area in contact with the enzyme solution has been previously established to achieve both high yield and a shorter incubation time in *Arabidopsis* [29]. Adapting the Arabidopsis-optimized tape method for cowpea overcomes major hurdles to achieving isolation efficiency as protoplast yields increase dramatically compared to the more commonly used leaf strip method [39].

The selection of plant tissue at the appropriate growth stage has also been previously reported as an important factor in protoplast isolation in multiple species [23, 28, 38, 40]. Results testing different plant ages showed that 10-12-day-old cowpea leaves produce a greater, more consistent yield and less debris than 6-7-day-old leaves. The 10-12-day-old leaves are neither floppy nor stiff, and the lower epidermis peels very easily with one pass of tape, reducing the possibility of tissue damage. Very young leaves are commonly recommended for protoplast isolations when using the leaf-cutting method [23, 41]. However, trials in this study found that leaves younger than six days old are not compatible with the use of the tape method. Younger leaves are too floppy when working with tape, and it is difficult to remove the lower epidermis without damaging the leaf. As avoiding mesophyll damage is a specific priority of the tape method, leaves younger than six days old are unsuitable for this method. Furthermore, previous publication in cowpea has reported that plants over 12 days old are not recommended for use in protoplast work, as older protoplasts have low survival rates during subsequent steps of the procedure [38]. From this information, it can be concluded that there is a limited window in which protoplast isolation conditions are optimal in a cowpea plant when using the tape sandwich method.

For the agroinfiltration experiments, the virulence of *Agrobacterium* strains varies from plant to plant, which might be due to the ability of the *Agrobacterium* to attach to the plant cells or T-DNA transfer mechanism [42, 43]. In this study, we found that EHA105-treated cowpea leaves showed higher GUS expression than the AGL-1 strain with the 35S promoter, while the CmYLCV promoter showed high levels of GUS expression with both *Agrobacterium* strains. We chose the 35S promoter for expression of Cas9 and used Cestrum Yellow Leaf Curling Virus promoter (CmYLCV) for gRNA expression to prevent the use of duplicate promoters. The CmYLCV promoter drives comparable or higher levels of expression than the 35S or maize (*Zea mays*) ubiquitin (ZmUbi) promoters in both dicots and monocots [44]. In this study, we also used Csy-type (CRISPR system yersinia) ribonuclease 4 (Csy4) for the expression of four gRNA simultaneously. It was reported that both Csy4 and tRNA expression systems are almost twice more effective than individual RNA polymerase III promoters systems in gRNAs expression and creating mutation, although the Csy4 expression system performed best regardless of the position of the gRNA in the array [34].

Finally, we tested our CRISPR-Cas9 vector containing designed sgRNAs in protoplasts by PEG mediated transformation and in cowpea leaves through agroinfiltration. In both methods, we found large deletions which covered all sgRNAs except sgRNA1. In summary, we have successfully established efficient protoplast transformation and agroinfiltration in cowpea as a testbed for optimizing genome editing using the CRISPR-Cas9 system by targeting the *PDS* gene.

## Supporting information

S1 Fig Schematic diagram of protoplast isolation for the leaf-cutting method (upper panel) and the tape sandwich method (lower panel). (PDF)

S1 Table Solutions used for protoplast isolation and transfection. (PDF)

S2 Table Primers used to amplify the cowpea PDS gene. (PDF)

S1 Raw images (PDF)
