# Supplementary material for: Optimization of gene editing in cowpea through protoplast transformation and agroinfiltration by targeting the phytoene desaturase gene
Source: PLoS One. 2023 Apr 5;18(4):e0283837. doi: 10.1371/journal.pone.0283837 (PMC10075407; doi:10.1371/journal.pone.0283837)
Supplement: S1 Fig — (PDF) [file pone.0283837.s001.pdf]

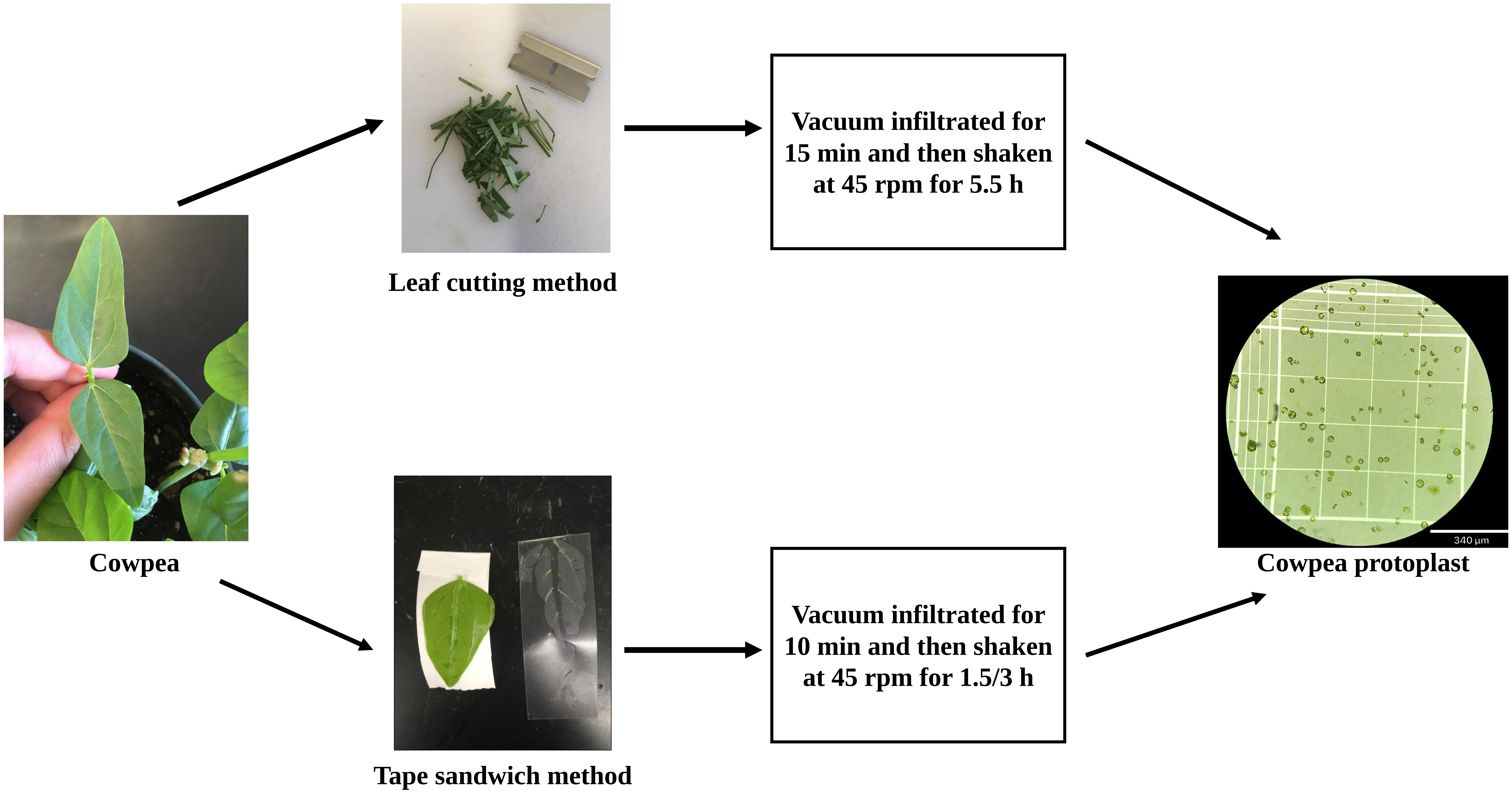

S1 Fig. Schematic diagram of protoplast isolation for the leaf-cutting method (upper panel) and the tape sandwich method (lower panel).
