# Supplementary material for: Optimization of gene editing in cowpea through protoplast transformation and agroinfiltration by targeting the phytoene desaturase gene
Source: PLoS One. 2023 Apr 5;18(4):e0283837. doi: 10.1371/journal.pone.0283837 (PMC10075407; doi:10.1371/journal.pone.0283837)
Supplement: S1 Table — (PDF) [file pone.0283837.s002.pdf]

S1 Table. Solutions used for protoplast isolation and transfection.

**Enzyme Solution (Freshly made)**	
**Cellulase R-10**	2% (w/v)
**Pectinase**	0.10%
**0.4% (w/v) Macerozyme R-10**	0.4% (w/v)
**Mannitol**	400 mM
**KCl**	20mM
**MES Buffer (pH 5.7)**	20mM
**deionized, distilled water**	up to 100ml% (w/v)
Heat at 55°C until dissolved, cool to room temperature	
**CaCl2**	10mM
**BSA**	0.1% (w/v)
Filter-sterilize with a 0.45 uM filter before use	
**Washing Solution (W5)**	
**NaCl**	154 mM
**$CaCl_2$**	125 mM
**KCl**	5 mM
**MES Buffer (pH 5.7)**	2 mM
**Glucose**	5 mM
**MMG Solution**	
**Mannitol**	400 mM
**$MgCl_2$**	15 mM
**MES Buffer (pH 5.7)**	4 mM
**deionized, distilled water**	
**40% PEG Solution (Freshly made)**	
**0.2 M Mannitol**	200 mM
**0.1 M $CaCl_2$**	100 mM
**PEG 4000**	40% (w/v)
Heat at 55°C until dissolved, cool to room temperature	
**Washing and Incubation Solution (WS1)**	
**Mannitol**	500 mM
**KCl**	20 mM
**MES Buffer (pH 5.7)**	4 mM
