# Supplementary material for: Optimization of gene editing in cowpea through protoplast transformation and agroinfiltration by targeting the phytoene desaturase gene
Source: PLoS One. 2023 Apr 5;18(4):e0283837. doi: 10.1371/journal.pone.0283837 (PMC10075407; doi:10.1371/journal.pone.0283837)
Supplement: S2 Table — (PDF) [file pone.0283837.s003.pdf]

S2 Table. Primers used to amplify the cowpea PDS gene.

Primer	Sequence (5' -> 3')
1 forward	AGAACCGAACACCCACGTTA
1 reverse	GTTGTAGTGGACGCGGAG
2 forward	ATTGCGTTGTCGTTTGGTGG
2 reverse	TGAGGAAATGAATGCGTAGCAG
3 forward	TATTTGGCTGATGCTGGGC
3 reverse	ACAGGACAGTTGCTAGACAG
4 forward	GCTATGCCAAGTAAACCCGG
4 reverse	GCATTAGGTCATGACTTGGC
5 forward	GGAACAACGAGATGCTGAC
5 reverse	GCAAGACAGACCATGAGAAGG
6 forward	TGCTGGTGAGGTGATTGAG
6 reverse	GCAAAATGAAATAATGGTGGCTG
7 forward	GATCGAGGGGGATGCTTACG
7 reverse	CTAACCCAGACCCCATGCAG
8 forward	CTGTTGTGGTGACATGCTGC
8 reverse	GGACGGCAAGGTTCACAATT
9 forward	TGAAGCCACGATGTCTGAGC
9 reverse	TGCTTAGATATGTGGCCGTC
