# Supplementary material for: Optimization of gene editing in cowpea through protoplast transformation and agroinfiltration by targeting the phytoene desaturase gene
Source: PLoS One. 2023 Apr 5;18(4):e0283837. doi: 10.1371/journal.pone.0283837 (PMC10075407; doi:10.1371/journal.pone.0283837)

S1 Raw Gel Image for Figure 3 (D) image 1

Lane 1 2 3 4 5 X

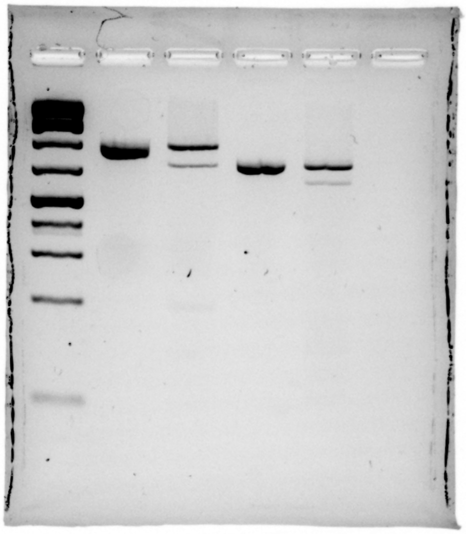

Gel image captured by Azure C200
gel documentation system

S1 Raw Gel Image for Figure 3 (D) image 2

Lane 6 7 8 9 10 X

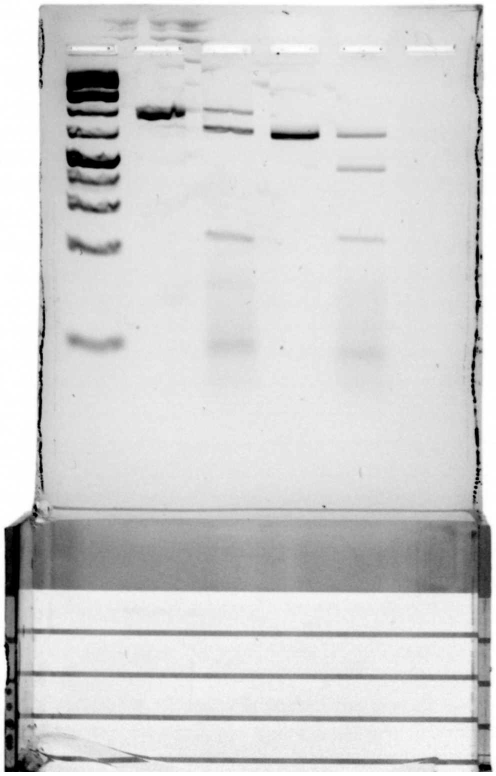

Supplement: S1 Raw images — (PDF) [file pone.0283837.s004.pdf]
